# Design of Quorum Sensing Inhibitor–Polymer Conjugates to Penetrate *Pseudomonas aeruginosa* Biofilms

**DOI:** 10.1021/acsmacrolett.2c00699

**Published:** 2023-02-15

**Authors:** Fadi Soukarieh, Pratik Gurnani, Manuel Romero, Nigel Halliday, Michael Stocks, Cameron Alexander, Miguel Cámara

**Affiliations:** † National Biofilms Innovation Centre, Biodiscovery Institute, University of Nottingham, Nottingham, NG7 2RD, United Kingdom; ‡ School of Life Sciences, Biodiscovery Institute, University of Nottingham, Nottingham, NG7 2RD, United Kingdom; § Division of Molecular Therapeutics and Formulation, Boots Science Building, School of Pharmacy, University of Nottingham, Nottingham, NG7 2RD, United Kingdom; ∥ Department of Microbiology and Parasitology, Faculty of Biology-CIBUS, Universidade de Santiago de Compostela, Santiago de Compostela, 15782, Spain; ⊥ School of Pharmacy, University of Nottingham Biodiscovery Institute, University of Nottingham, Nottingham, NG7 2RD, United Kingdom

## Abstract

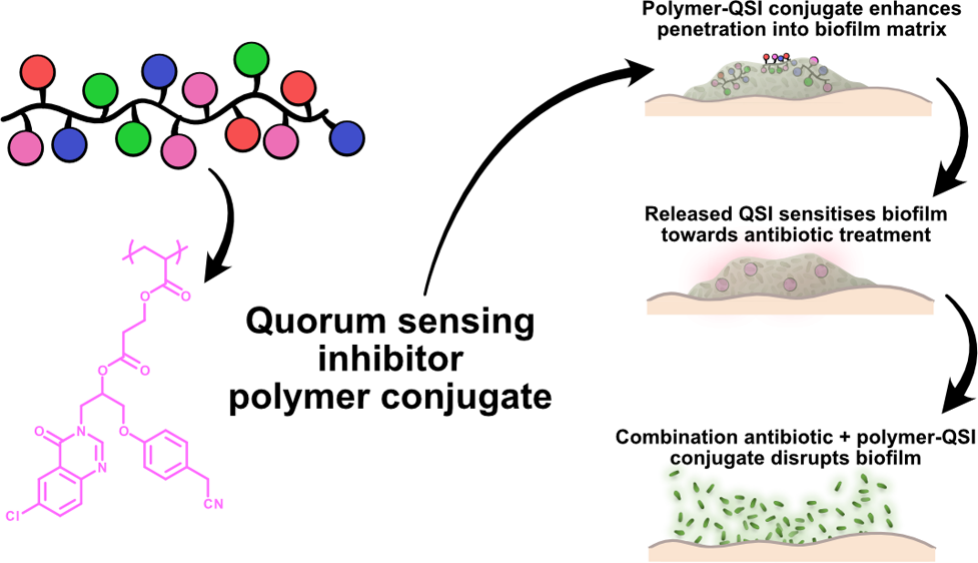

Antimicrobial resistance (AMR) is a global threat to public health with a forecast of a negative financial impact of one trillion dollars per annum, hence novel therapeutics are urgently needed. The resistance of many bacteria against current drugs is further augmented by the ability of these microbes to form biofilms where cells are encased in a slimy extracellular matrix and either adhered to a surface or forming cell aggregates. Biofilms form physiochemical barriers against the penetration of treatments such as small molecule antibacterials, rendering most treatments ineffective. *Pseudomonas aeruginosa*, a priority pathogen of immediate concern, controls biofilm formation through multiple layers of gene regulation pathways including quorum sensing (QS), a cell-to-cell signaling system. We have recently reported a series of inhibitors of the PqsR QS regulator from this organism that can potentiate the action of antibiotics. However, these QS inhibitors (QSIs) have shown modest effects on biofilms in contrast with planktonic cultures due to poor penetration through the biofilm matrix. To enhance the delivery of the inhibitors, a small library of polymers was designed as carriers of a specific QSI, with variations in the side chains to introduce either positively charged or neutral moieties to aid penetration into and through the *P. aeruginosa* biofilm. The synthesized polymers were evaluated in a series of assays to establish their effects on the inhibition of the Pqs QS system in *P. aeruginosa*, the levels of inhibitor release from polymers, and their impact on biofilm formation. A selected cationic polymer–QSI conjugate was found to penetrate effectively through biofilm layers and to release the QSI. When used in combination with ciprofloxacin, it enhanced the biofilm antimicrobial activity of this antibiotic compared to free QSI and ciprofloxacin under the same conditions.

Most pathogenic bacteria can form highly resistant communities known as “biofilms” consisting of cell clusters surrounded by a viscous hydrated extracellular matrix. The cooperative microbial environment within a biofilm is now recognized as one of the main reasons for the clinical failure of antibiotic treatment, particularly in chronic nosocomial infections.^1,2^ The enhanced antimicrobial tolerance of bacterial biofilms^3^ is attributed to (i) the physicochemical properties of extracellular polymeric substances (EPS) produced by the bacteria, which can act as barriers to retard or repel antimicrobial penetration within the biofilm,^4^ and (ii) the physiological status of cells using genes involved in antibiotic resistance, i.e., those coding for drug efflux pumps, which can be highly expressed in these communities.^5^ Eradication of such biofilm infections is typically only achievable with prolonged high doses of multidrug treatments, which pose toxicity and antimicrobial resistance risks, or invasive surgical removal of infected tissue.^6^ Hence, there is an urgent need to develop new therapeutic strategies that improve the efficacy of conventional antimicrobial treatments toward bacterial biofilms while simultaneously mitigating the current toxicity and resistance threats.

In particular, biofilm-centered infections caused by the bacterial pathogen *Pseudomonas aeruginosa* are a significant threat to vulnerable patients such as those with cystic fibrosis, chronic wounds, chronic obstructive pulmonary disease and urinary tract infections.^7^ Biofilm formation in *P. aeruginosa* is tightly regulated by complex regulatory networks that integrate intra- and extracellular signaling systems controlling the expression of genes involved in this process.^7−9^ One of these signaling systems is known as quorum sensing (QS), which involves small chemical signals known as autoinducers that govern the expression of diverse genes in a population density-dependent manner.^9^ It has been well established that QS allows clustered bacteria to express specific traits that promote antimicrobial tolerance and enable “community control” over immune evasion, expression of drug efflux pumps, and even cell death.^3,10−13^ Given this, the development of chemical quenchers for QS, known as quorum sensing inhibitors (QSIs), which can compete with endogenous signals to suppress virulence factor expression and sensitize biofilms toward antibiotic treatment, has been a significant focus to combat pathogenic biofilms.^15−19^ However, as with conventional antibiotics, the efficacy of QSIs depends on their biofilm penetration properties to reach individual cells.^20^

We previously reported a high-throughput chemical and *in silico* screening of inhibitors for the *Pseudomonas* Quinolone Signal (PQS) system, one of three interconnected autoinducing QS systems of *P. aeruginosa*, resulting in the discovery of several potent QSIs that could attenuate the production of virulence traits that play a key role in the disease process.^15,16,19^ However, we found *in vitro* that their efficacy was substantially reduced against biofilms of this bacterium, due most likely to poor penetration through the matrix. Therefore, in this study we hypothesized that we could enhance the activity of a specific QSI, (*R*)-2-(4-(3-(6-chloro-4-oxoquinazolin-3(4*H*)-yl)-2-hydroxypropoxy)phenyl)acetonitrile, by conjugating it to a carrier, thus improving delivery throughout the biofilm and enabling potentiation of antibiotic treatment against sessile *P. aeruginosa* communities.^20^

Synthetic polymers that mimic the structure and function of antimicrobial peptides, but which can be made at scale, are promising for a range of antimicrobial and antibiofilm applications.^21,22^ These polymers typically incorporate cationic and hydrophobic functional groups for synergistic bacterial membrane attraction and disruption.^23−26^ A number of other polymers with a variety of side-chain functionalities and macromolecular architectures that exhibit activity against biofilms have been identified through high-throughput or design-led studies.^25,27^ Many of these materials have also been utilized for successful co-delivery of antimicrobial agents (e.g., penicillins, aminoglycosides, nitric oxide) or for modulation of bacterial aggregation or dispersal through QS.^28^

Encouraged by these pioneering studies and our previous experience with QSI nanoparticle delivery vectors,^20^ here we report the development of a novel PQS QSI polymer conjugate system, which displays efficient permeation through the biofilm matrix and enhances ciprofloxacin activity against *P. aeruginosa* biofilms (Figure 1). We describe the design and synthesis of novel polymer–QSI conjugates as a delivery system to enhance QSI bioavailability and show their effects on highly resistant bacterial cells encased in mature clinically relevant biofilms, achieving virulence inhibition and enhancing the antibiotic effects on a life-threatening pathogen.

**Figure 1:**
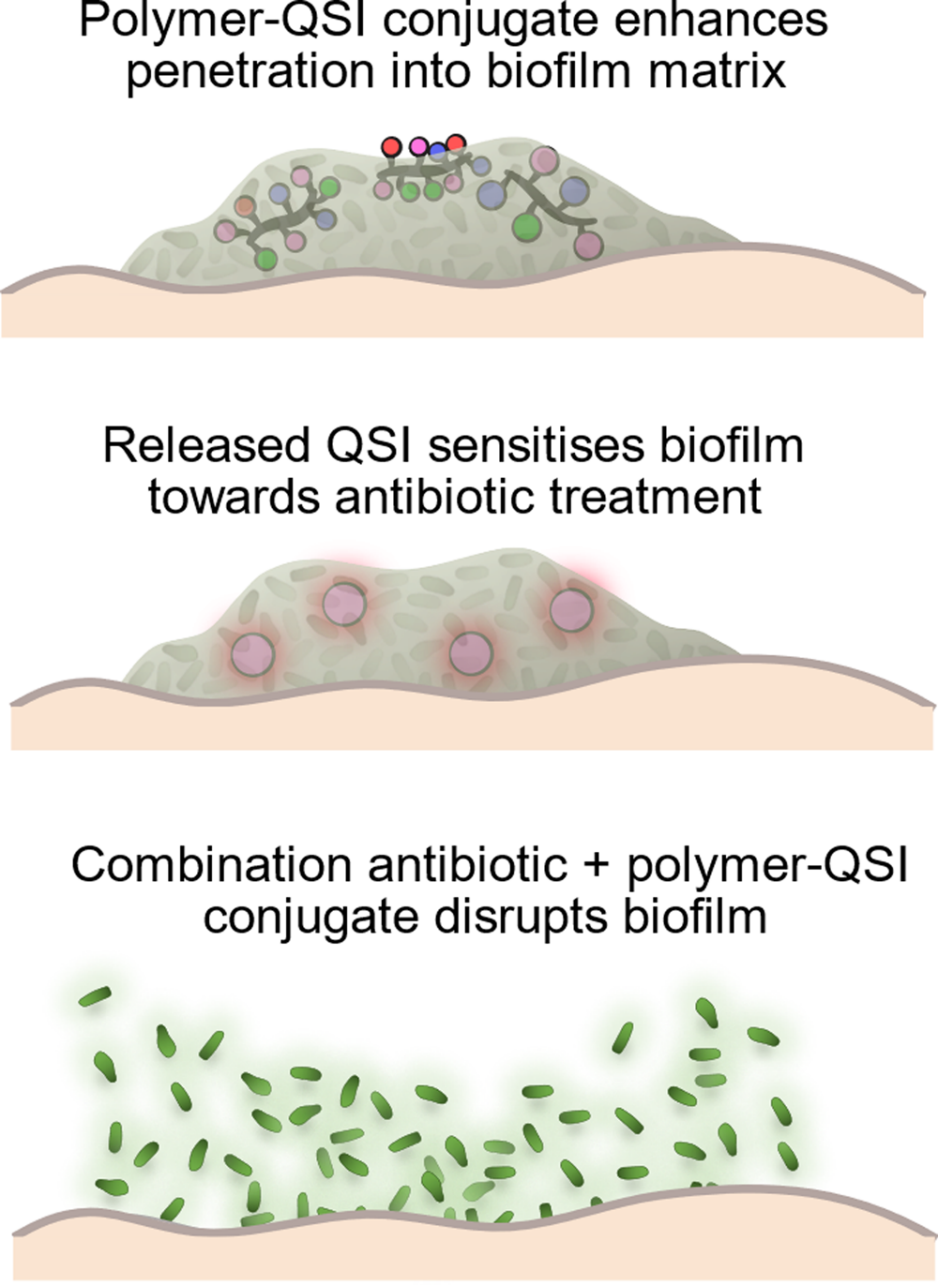
Polymer–QSI conjugate concept for enhanced QSI delivery and improved antibiotic performance.

For our polymer structures, we built on previous reports by Boyer and co-workers who utilized high-throughput screens or “hypothesis-led” libraries to screen hydrophobic and cationic monomer composition, polymer topology, and molar mass to optimize for biocompatibility and potential permeability through thick biofilms.^29−33^ From these previous findings we therefore postulated that such polymer structures might also possess efficient biofilm permeation, hence they could be excellent candidates for QSI incorporation.

To control the content and distribution of our chosen QSI within the polymer chain, we initially synthesized a novel QSI monomer, QSI acrylate (QSIA), by coupling the alcohol functionality of QSI with 2-carboxyethyl acrylate using conventional EDC coupling. It has been previously shown that the activity of this QSI is dependent on the free hydroxyl group at the β-amino alcohol linker position, therefore this specific structure was chosen to provide separation of the QSI from the polymer backbone that could then be accessed by biofilm esterases or hydrolysis for release. This design choice also means that the QSI molecule will be inactive when conjugated, creating a pro-QSI polymer conjugate system. The QSIA was copolymerized with poly(ethylene glycol) acrylate (PEGA), 2-ethylhexyl acrylate (EHA), and 2-hydroxyethyl acrylate (HEA) as a neutral comonomer or 2-dimethylaminoethyl acrylate (DMAEA) as cationic comonomer via RAFT polymerization to prepare P1-QSI and P2-QSI, respectively (Figure 2). The target monomer compositions were chosen following the previous studies by Boyer and co-workers,^30^ with a 10%:30%:10%:50% molar ratio of EHA:PEGA:QSIA:HEA/DMAEA. QSI polymer conjugates were synthesized at 70 °C with PABTC as the RAFT agent and ACVA as the thermal initiator with a target chain length of 125, reaching ∼80% monomer conversion after 5 h, yielding P1-QSI and P2-QSI, respectively, with an approximate DP of 100 (Table S1). For non-QSI controls, we also prepared an analogous pair of HEA and DMAEA containing copolymers without QSIA, and utilized benzyl acrylate (BzA) as a chemically similar but nonactive QSI monomer at the same ratios and under the same conditions described above to yield P1 and P2 as non-QSI bearing copolymer controls. All polymers displayed a similar comonomer composition to their targeted structure via ^1^H NMR spectroscopy (Figure S1) and narrow molecular weight distribution (*Đ* < 1.5) via DMF-SEC (Figure S2 and Figure 2, inset table). Although the DMF-SEC molar masses deviated from the theoretical molar masses, this is attributed to the difference in hydrodynamic volume between our copolymers and the PMMA standards used for conventional calibration. This effect may be particularly pronounced given that the brush-like PEGA monomer, which contributes to a large proportion of the total polymer mass, will have substantially different solvation to PMMA in DMF.

**Figure 2:**
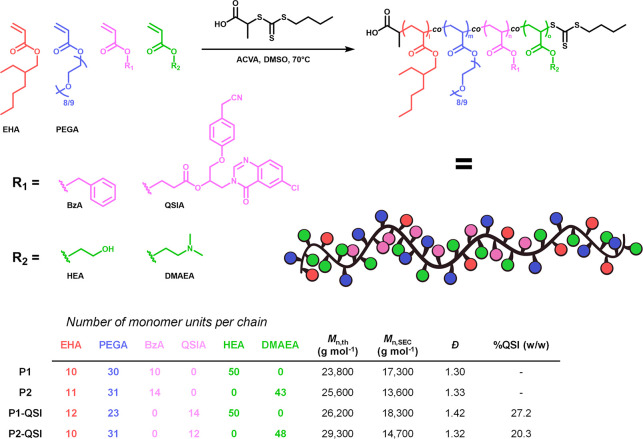
Synthetic scheme for RAFT polymerizations to prepare control polymers P1 and P2, and QSI polymer conjugates, P1-QSI and P2-QSI. Inset table describes the number of each monomer unit per chain and QSI loading determined by ^1^H NMR spectroscopy, theoretical molar masses determined using eq 1 (Figure S2), and experimental molar masses and *Đ* through DMF-SEC.

To investigate the ability of the synthesized polymers to interfere with the Pqs system in PAO1-L, inhibition of a P_*pqsA*_-*lux* transcriptional fusion, which reports the PQS-dependent activation of the *pqs* operon mediated by PqsR, the LysR-type receptor of PQS,^16^ was assessed. Pharmacological inhibition of PqsR prevents the formation of the PqsR–PQS complex, leading to less activation of the *pqsA* promotor and the consequent disruption of the transcription of downstream genes. Hence, the successful release of QSI causes a decrease in the *lux* operon expression, leading to lower luminescence values. The QSI polymers (P1-QSI and P2-QSI) were tested at concentrations of 50 μM against a positive control of QSI (10 μM), their polymer control counterparts (P1 and P2), and a negative control of solvent vehicle (0.1% DMSO).

As expected, control polymers P1 and P2 displayed no QS inhibition activity in the reporter assay compared to the 0.1% DMSO control. In addition, these polymers were shown to not affect bacterial growth (Figures 3A,D and S3.A). Despite bearing QSIA monomer units, P1-QSI, containing noncharged comonomers, exhibited no improved QS inhibition compared to P1. In contrast, P2-QSI, decorated with cationic DMAEA units, caused a 70% reduction in luminescence after a 24 h incubation compared to its control polymer without QSI. The reduction in QS activity in response to P2-QSI was also found to be concentration responsive, with limited inhibition at 0.5 μM and 5 μM, but a significant reduction in luminescence was observed at 50 μM relative QSI concentration (Figure S3.B). Notably, none of the materials exhibited any significant growth inhibitory activity toward the *P. aeruginosa* culture, suggesting any difference in luminescence observed was solely due to the QSI activity (Figure 3B,D).

**Figure 3:**
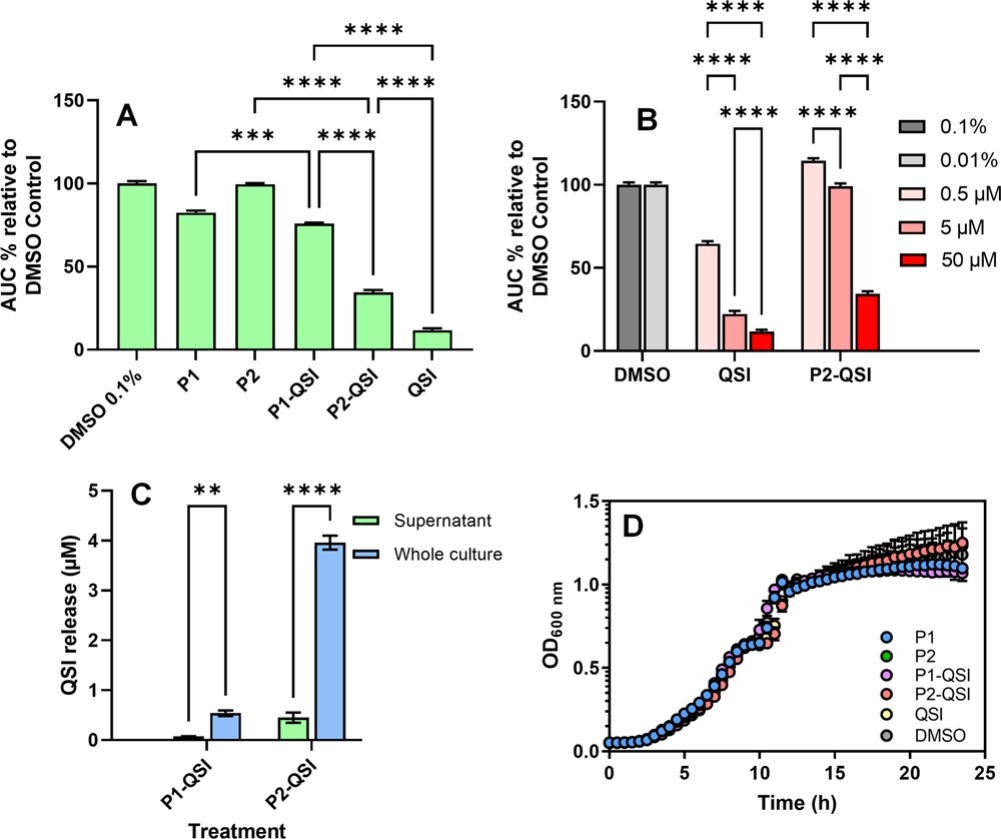
Evaluation of QSI polymers: (A) Comparison of the activity of polymers (control: P1, P2; QSI: P1-QSI, P2-QSI), QSI, and DMSO control in the bioreporter assay using PAO1-L mCTX::P_*pqsA*_-*lux* at a concentration of 50 μM for polymers and 10 μM for QSI; (B) Comparison of activity P2-QSI at different concentrations; (C) LCMS/MS analysis study of QSI release in the supernatant and whole cell culture of PAO1-L; (D) Growth curves of PAO1-L in the presence of 50 μM of polymers or 10 μM of QSI compared to a control of DMSO 0.1%. For A and B, the area under the curve (AUC) corresponds to relative light units divided by OD_600_. Statistical analysis was performed using a one-way ANOVA and Tukey posthoc test. Bars represent mean ± SD for *n* = 3. Asterisks represent statistical significance: *p < 0.05, **p < 0.01, ***p < 0.001, ***p < 0.0001.

Although in this setting our QSI polymer conjugates did not outperform the native QSI molecular control, we hypothesized that this might be due to a difference in the bioavailability of the unconjugated QSI in the bacterial culture compared to the polymer conjugates. To examine this, P1-QSI and P2-QSI (equivalent 50 μM QSI dose) were incubated with a *P. aeruginosa* culture at 37 °C to measure the quantity of released QSI in both the whole bacterial culture (cell fraction + supernatant) and supernatant only. Interestingly, with P1-QSI, both the cell and supernatant fractions exhibited poor QSI availability (<1 μM) compared to P2-QSI that displayed a ∼ 5-fold increase in QSI concentration (∼4 μM, 8% release), particularly in the cell fraction (Figure 3C). These findings may also explain the improved performance of P2-QSI to disrupt the PQS system, suggesting that this analogue may accelerate the hydrolysis of the QSI-ester bond while also improving the association with bacteria through the net positive charge via the DMAEA units. This accelerated release is consistent with other studies using such polymer systems supporting that the local tertiary amine concentration may accelerate release of complexed or conjugated materials, such as siRNA, via a self-catalyzed process.^34^ We therefore believe this is the major driving force of the enhanced release of the QSI from P2-QSI compared to P1-QSI where the DMAEA monomer is not present. These data also explains the findings observed in the concentration dependency experiment (Figure 3B), which show roughly equivalent quorum sensing inhibition between a 5 μM dose of QSI and 50 μM dose of P2-QSI (equivalent to 4 μM released QSI). Moreover, this suggests that maximizing the release of the QSI molecule from the polymer conjugate is imperative in further improving the performance of these materials.

Encouraged by our results with P2-QSI, we evaluated the diffusion of the P2-QSI polymer through the biofilm matrix. PAO1-L biofilms (2-day old, ∼ 60 μm depth) were exposed to a Rhodamine B labeled analogue of P2-QSI conjugate, and the fluorescence signal was measured at different layers of the biofilm depth using confocal microscopy and image analysis. After 12 h incubation, the dye-labeled P2-QSI showed complete penetration throughout the biofilm matrix (Figure 4A), confirming the ability of the conjugate to diffuse into PAO1-L biofilms effectively.

**Figure 4:**
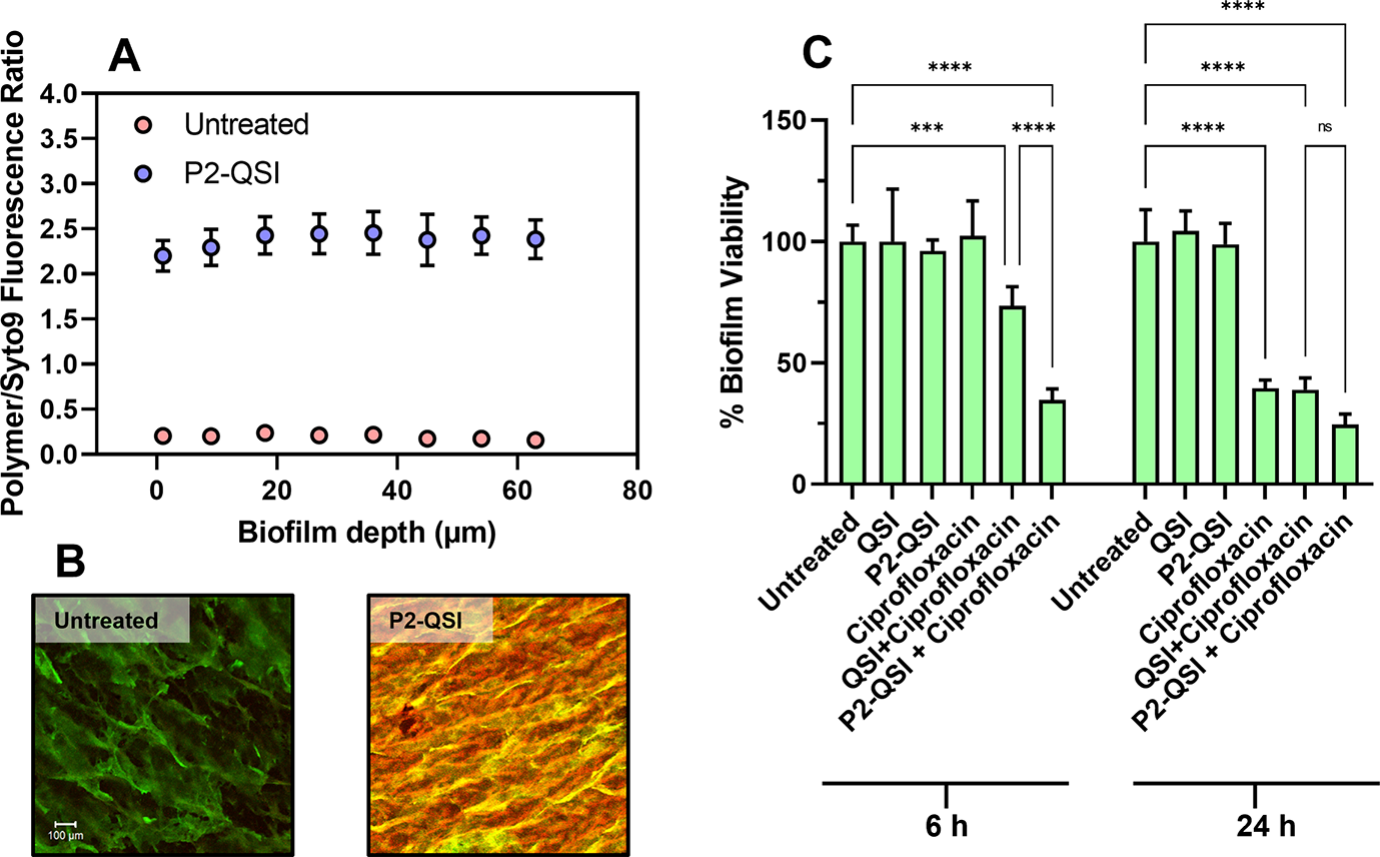
(A) Penetration profile of the rhodamine B-labeled P2-QSI polymer within 12 h of incubation with mature *P. aeruginosa* PAO1-L biofilms (2-day-old). The fluorescence intensities of the P2-QSI conjugate are normalized to the background fluorescence of the biofilm stain (SYTO9) at different depths. (B) Representative confocal laser scanning microscopy (CLSM) images of PAO1-L biofilms after 2 days of growth and 12 h of exposure to the P2-QSI polymer labeled with rhodamine B. Rhodamine B fluorescence signal is shown in red and the bacteria are stained green with SYTO9 dye. Scale bar: 100 μm. (C) Bar charts showing viability in PAO1-L biofilms quantified after treatment with different conditions for 6 and 24 h. The concentrations of the drugs used were ciprofloxacin-60 μg mL^–1^, QSI (10 μM), and P2-QSI (100 μM). Statistical analysis was performed using a one-way ANOVA and Tukey posthoc test. Bars represent mean ± SD for *n* = 3. Asterisks represent statistical significance: *p < 0.05, **p < 0.01, ***p < 0.001, ***p < 0.0001.

To assess whether the P2-QSI conjugate assisted in the delivery of the PqsR antagonist into mature biofilms and demonstrate the impact of this antivirulence compound in combination therapy, PAO1-L biofilms were treated with P2-QSI polymer with or without combination with ciprofloxacin. Results showed that, in common with planktonic cells, PAO1-L biofilms remained unaltered upon exposure to P2-QSI or free QSI, both after short (6 h) and long (24 h) exposures, suggesting that the polymer and Pqs system inhibitor inflicted no adverse effect on bacterial viability (Figure 4). On the contrary, when the biofilms were challenged with a high dose of ciprofloxacin (×300 MIC), a decrease in biofilm viability (∼60%) was recorded, but only after a 24 h exposure showing the tolerance of these communities against antimicrobials under the tested conditions. Interestingly, biofilms treated with ciprofloxacin in combination with free QSI resulted in enhanced cell killing after 6 h. However, the most effective treatment was administering the P2-QSI polymer along with ciprofloxacin, as a noticeable improvement in the treatment of the biofilms was observed, especially after a short time of treatment exposure (∼70% reduction in viability).

This outcome indicates that the bacterial viability was likely affected because of the deeper penetration of the P2-QSI polymer and the effective delivery of the PQS antagonist *in situ* to render the biofilm sensitive to the killing action of ciprofloxacin. Therefore, the evidence presented here suggests that the hydrophobic nature of PqsR antagonists and the limited efficacy of antibiotics against biofilms require more effective carriers for biofilm penetration and release of antimicrobial compounds, and the polymers developed in this study could successfully mediate these adjuvant therapies.

Herein we report a new polymer design to aid the penetration and delivery of our antibiotic adjuvant QSI throughout *P. aeruginosa* biofilms. As previously reported, (R)-2-(4-(3-(6-chloro-4-oxoquinazolin-3(4H)-yl)-2-hydroxypropoxy)phenyl)acetonitrile is a novel QSI that can disrupt cell-to-cell communication at the level of the Pqs system, leading to a reduction in *P. aeruginosa* phenotypes such as pyocyanin production at a concentration of 3 μM. However, the free QSI itself demonstrated modest activity on *P. aeruginosa* biofilms, possibly due to poor penetration through the extracellular biofilm matrix. To overcome this, polymers were designed and synthesized to integrate a QSI monomer and either a DMAEA positively charged comonomer or HEA neutral moiety. These polymers, along with controls, were initially assessed using a luminescence bioreporter assay to evaluate the release and activity of QSI compared to unpolymerized control. The experiment concluded that P2-QSI showed superior activity to P1-QSI, nevertheless, it was lower than the free QSI, possibly due to a difference in bioavailability. Furthermore, the discrepancy in activity between P1-QSI and P2-QSI-62 was related to the rate of release of QSI, as shown by LCMS/MS analysis. It was therefore hypothesized that DMAEA might enhance the rate of ester bond hydrolysis and QSI release. Subsequently, the P2-QSI polymer was labeled with rhodamine to assess its penetration in *P. aeruginosa* stained with Syto-9 to reveal that this polymer is able to penetrate deeply throughout the biofilm matrix. Moreover, the effect of ciprofloxacin on biofilm viability was significantly increased when combined with P2-QSI compared to unpolymerized QSI, particularly in the early hours of treatment.

## Supporting Information Available

Details about the synthetic and characterization methodology of the QSI monomer and polymers, microbiological evaluation experiments, and biofilm assays (PDF)
