# Supplementary material for: Design of Quorum Sensing Inhibitor–Polymer Conjugates to Penetrate Pseudomonas aeruginosa Biofilms
Source: ACS Macro Lett. 2023 Feb 15;12(3):314–9. doi: 10.1021/acsmacrolett.2c00699 (PMC10035027; doi:10.1021/acsmacrolett.2c00699)
Supplement: Supplementary file 1 — mz2c00699_si_001.pdf [file mz2c00699_si_001.pdf]

Supporting Information for:

Design of quorum sensing inhibitor-polymer conjugates to penetrate *Pseudomonas aeruginosa* biofilms

Fadi Soukarieh[1,2†*], Pratik Gurnani[3†*], Manuel Romero[1,4†*], Nigel Halliday[2], Michael Stocks[5], Cameron Alexander[3] and Miguel Cámara[1,2]

†These authors have equal contributions.

*Corresponding authors

Affiliations

[1] National Biofilms Innovation Centre, Biodiscovery Institute, University of Nottingham, Nottingham, NG7 2RD, UK.

[2] School of Life Sciences, Biodiscovery Institute, University of Nottingham, Nottingham, NG7 2RD, UK.

[3] Division of Molecular Therapeutics and Formulation, Boots Science Building, School of Pharmacy, University of Nottingham, Nottingham, NG7 2RD, UK.

[4] Department of Microbiology and Parasitology, Faculty of Biology-CIBUS, Universidade de Santiago de Compostela, Santiago de Compostela, 15782, Spain.

[5] School of Pharmacy, University of Nottingham Biodiscovery Institute, University of Nottingham, Nottingham, NG7 2RD, UK.

*Corresponding Authors Emails: fadi.soukarieh@nottingham.ac.uk, pratik.gurnani@nottingham.ac.uk, manuelromero.bernardez@usc.es

Materials

2-Hydroxyethyl acrylate (HEA, 96%), 2-ethylhexyl acrylate (EHA, 98%), 2-(dimethylamino)ethyl acrylate (DMAEA, >98%), 1-carboxyethyl acrylate (CEA) and benzyl acrylate (BzA, >99%) were obtained from Sigma-Aldrich and the inhibitor removed by passing the monomers through a column of basic aluminium oxide. DMSO-d_6 (99.5% D atom), was obtained from Sigma Aldrich and used for ^{1}H NMR spectroscopy. Thermal initiator 4,4′-Azobis(4-cyanovaleric acid) (ACVA, >98%) was purchased from Sigma Aldrich and used as received. RAFT agent propionic acid *n*-butyl trithiocarbonate (PABTC) was synthesised as previously reported.[1] Solvents and other reagents were acquired from commercial sources and used as received unless stated otherwise.

Methods

Instrumentation and Analysis

NMR spectroscopy

^{1}H NMR and ^{13}C NMR spectra were recorded on a Bruker DPX-400 spectrometer using deuterated solvent (materials section).

Size exclusion chromatography

A Polymer Laboratories PL-50 instrument equipped with differential refractive index (DRI) was used for SEC analysis. The system was fitted with 2 x PLgel Mixed D columns (300 x 7.5 mm) and a PLgel 5 µm guard column. The eluent used was DMF with 0.1% LiBr. Samples were run at 1 min^{-1} at 50°C. Poly(methyl methacrylate) standards (Agilent EasyVials) were used for calibration between 955,500 – 550 g mol^{-1}. Analyte samples were filtered through a membrane with 0.22 µm pore size before injection. Respectively, experimental molar mass ($M_{n,SEC}$) and dispersity ($Đ$) values of synthesised polymers were determined by conventional calibration using Cirrus GPC software.

Theoretical molar mass calculation

$$M_{n,th} = \frac{[M]_0 \, p M_M}{[CTA]_0} + M_{CTA}$$

Equation 1 Calculation of theoretical number average molar mass ($M_{n,th}$) where $[M]_0$ and $[CTA]_0$ are the initial concentrations (in mol dm^{-3}) of monomer and chain transfer agent respectively. p is the monomer conversion as determined by ^{1}H NMR spectroscopy. M_M and M_{CTA} are the molar masses (g mol^{-1}) of the monomer and chain transfer agent respectively.

Synthesis and characterisation

QSI (3-((1-(6-chloro-4-oxoquinazolin-3(4H)-yl)-3-(4-(cyanomethyl)phenoxy)propan-2-yl)oxy)-3-oxopropyl acrylate) synthesis

To a stirred solution of QSI (1 g, 2.7 mmol) and 1-carboxyethyl acrylate (0.5 g, 3.5 mmol) in dichloromethane (70 mL), EDCI.HCl (0.54 g, 3.5 mmol) and DMAP (0.033 g, 0.27 mmol) were added and the resulting mixture was stirred for 16 h. Subsequently, the reaction mixture was washed with water and then washed with saturated sodium bicarbonate solution and the organic layer was dried over granular magnesium sulphate. The organic layer was then reduced, and the crude residue was purified using flash chromatography using Hexane:Ethyl Acetate 8:2 to afford the product as a white solid (0.74 g, 55%).

^{1}H NMR (400 MHz, Chloroform-*d*) δ 8.26 (d, J = 2.3 Hz, 1H), 8.08 (s, 1H), 7.76 – 7.62 (m, 2H), 7.30 – 7.23 (m, 2H), 6.96 – 6.87 (m, 2H), 6.47 – 6.27 (m, 1H), 5.99 (dd, J = 17.3, 10.4 Hz, 1H), 5.75 (dd, J = 10.4, 1.4 Hz, 1H), 5.57 (dq, J = 7.8, 3.9 Hz, 1H), 4.68 – 4.57 (m, 1H), 4.49 – 4.08 (m, 5H), 3.71 (s, 2H), 2.79 – 2.63 (m, 2H). LCMS *m/z* calculated for $C_{25}H_{22}ClN_3O_6$ $[M]^+$: 495.1, found 495.7 ^{13}C NMR (101 MHz, DMSO-d_6) δ 171.59, 167.35, 159.57, 158.37, 149.16, 147.44, 134.72, 131.11, 126.12, 123.02, 119.52, 115.31, 69.23, 68.88, 61.12, 49.77, 35.75, 24.25.

Polymer synthesis

Polymers were synthesised using the following procedure, utilising the conditions described in Table S1. PABTC, monomers (in the appropriate ratios) and ACVA (from a pre-made stock solution in DMSO) were dissolved in DMSO. The solution was fitted with an appropriately sized rubber septum and purged with nitrogen for 20 min. The polymerisation solution was subsequently immersed in an oil bath preheated to 70°C until the polymerisation reached 80-90% monomer conversion as determined by ^{1}H NMR spectroscopy. The reaction was cooled to ambient temperature and opened to oxygen to quench the polymerisation. Polymers were diluted 3-fold in acetone and purified by precipitation twice in diethyl ether (20-fold volume). The precipitated polymer was dissolved in dichloromethane (DCM), dried under reduced pressure and residual solvents were removed in a vacuum oven at 40°C for 48 h. Fluorescent analogues were synthesised using the same conditions with 1 mol% of acryloxyethyl thiocarbamoyl rhodamine B relative to the total monomer concentration (~1 fluorescent dye per polymer chain).

Microbiological experiments

Biosensor reporter assay:

To evaluate the activity of QSI-polymer conjugates, the reporter strain *P. aeruginosa* PAO1-L *mCTX*::P$_{pqsA}$-*lux* was used as previously described.[2] For testing, the polymers were assessed at the specified concentrations prepared from 50 mM DMSO stocks.

QSI acrylate (QSIA) release

Stock solutions of polymers P1-QSI and P2 were prepared in DMSO at an equivalent concentration of 50 mM of QSI. A 2 mL LB culture of PAO1-L was grown overnight at 37° with shaking at 200 rpm. Then 10 mL aliquots of fresh LB, with polymer P1-QSI or P2-QSI added at an equivalent concentration of 50 µM, were inoculated with 10 µL of the overnight culture and incubated for 17 h at 37°C with shaking at 200 rpm. Negative control samples were prepared in an identical manner but without the addition of bacterial inoculum. Samples of each growth condition were prepared in triplicate. Sterile-filtered supernatant samples and whole culture samples were collected and stored at -20°C until required for analysis. For analytical sample preparation, 10 µL of supernatant samples were diluted with 90 µL of MeOH. Whole culture samples were prepared by spiking 10 µL of the whole culture into 1 mL of water and extracting each cell suspension three times with 0.5 mL of EtOAc. For each sample, combined organic extracts were dried under vacuum and redissolved in 100 µL of 10% (v/v) water in MeOH. Analysis of the prepared samples was conducted by LC-MS/MS. A Shimadzu series 10AD VP LC system was used in tandem with an Applied Biosystems Qtrap 4000 hybrid triple-quadrupole linear ion trap mass spectrometer equipped with an electrospray ionisation interface. Chromatography was achieved with a Phenomenex Gemini C18 column (3.0 µm, 100 x 3.0 mm) using a mobile phase of 0.1% (v/v) formic acid (A) and 0.1% (v/v) formic acid in methanol (B), at a constant flow rate of 450 µL/min. Analytes were eluted with a gradient mobile phase, starting at 10% B, increasing linearly to 99% B over 5.5 min. After 1.5 min at this gradient the mobile phase was returned immediately to 10% B. Total run time was 10 min per sample. The analysis was conducted with the MS operating in MRM (multiple reaction monitoring) mode under positive electrospray conditions, screening the LC eluent for QSI using a parent-product ion mass transition of *m/z* 370.1181.

Biofilm viability and polymer penetration studies

Mature 2-day-old PAO1-L biofilms were used to characterise the effect of P2-QSI polymer and ciprofloxacin treatment combinations. Biofilms were grown on round glass coverslips (13 mm Ø, #1.5 thickness) under dynamic conditions (20 rpm) in FAB 10 mM glucose medium with or without supplementation with P2-QSI (100 µM), inoculated with diluted (OD_{600nm} = 0.01) bacteria from overnight cultures in LB. The biofilms were cultivated at 30°C for 2 days with medium replacement after 24h incubation, then washed in PBS to remove loosely attached cells and incubated for a further 6 or 24 h in fresh medium supplemented with various treatments. These included free ciprofloxacin 60 µg/mL (x300 the MIC of planktonic *P. aeruginosa* cells)[3], QSI at 10 µM and ciprofloxacin in combination with QSI. Biofilms exposed to each treatment were washed in PBS and the viability of attached cells was evaluated by fluorescent staining using the LIVE/DEAD® BacLight™ Bacterial Viability kit (Molecular Probes, Life Technologies) according to manufacturer instructions. Following staining, coverslips were rinsed with distilled water and imaged using a LSM700 AxioObserver (Carl Zeiss, Germany) confocal laser scanning microscope (CLSM). Viable and non-viable biofilm biomass quantification from image stacks of biofilms was done with Fiji-ImageJ software. Live/dead ratios were established for each treatment and compared to untreated controls.

In parallel, and to ascertain polymer diffusion through the biofilm matrix, a Rhodamine B labelled analogue of P2-QSI conjugate was also tested against 2-day-old PAO1-L biofilms. After 12 h of incubation with the fluorescent polymer at 50 µM, biofilm samples were collected and stained with Syto9 fluorescent dye prior to CLSM image acquisition to simultaneously detect bacterial cells (green fluorescence) and P2-QSI polymer (red fluorescence). P2-QSI diffusion was assessed by quantifying the Rhodamine signal associated with each image stack and normalised to the biofilm biomass at different depths.

Statistical analysis

Graphical representations and statistical analysis were performed in GraphPad Prism, version 8. Statistical differences were analysed using either multiple t-tests adjusted for multiple comparisons or one-way ANOVA with multiple comparisons, with $p<0.05$ used to indicate significance.

Supporting information:

Table S1 RAFT polymerisation conditions for all synthesised polymers. Polymerisations were conducted in DMSO as solvent, total $[M]_0$ = 3 M, PABTC as RAFT agent targeting a DP of 125 ($[M]_0/[CTA]_0$ = 125), ACVA as initiator ($[CTA]_0/[I]_0$ = 8) and polymerisations proceeded for 5 h at 70°C. [a]Determined by ^{1}H NMR spectroscopy.

	[EHA] (M)	[PEGA] (M)	[BzA] (M)	[QSIA] (M)	[HEA] (M)	[DMAEA] (M)	Conversion (%)[a]
P1	0.3	0.9	0.3	0	1.5	0	77
P2	0.3	0.9	0.3	0	0	1.5	76
P1-QSI	0.3	0.9	0	0.3	1.5	0	75
P2-QSI	0.3	0.9	0	0.3	0	1.5	81

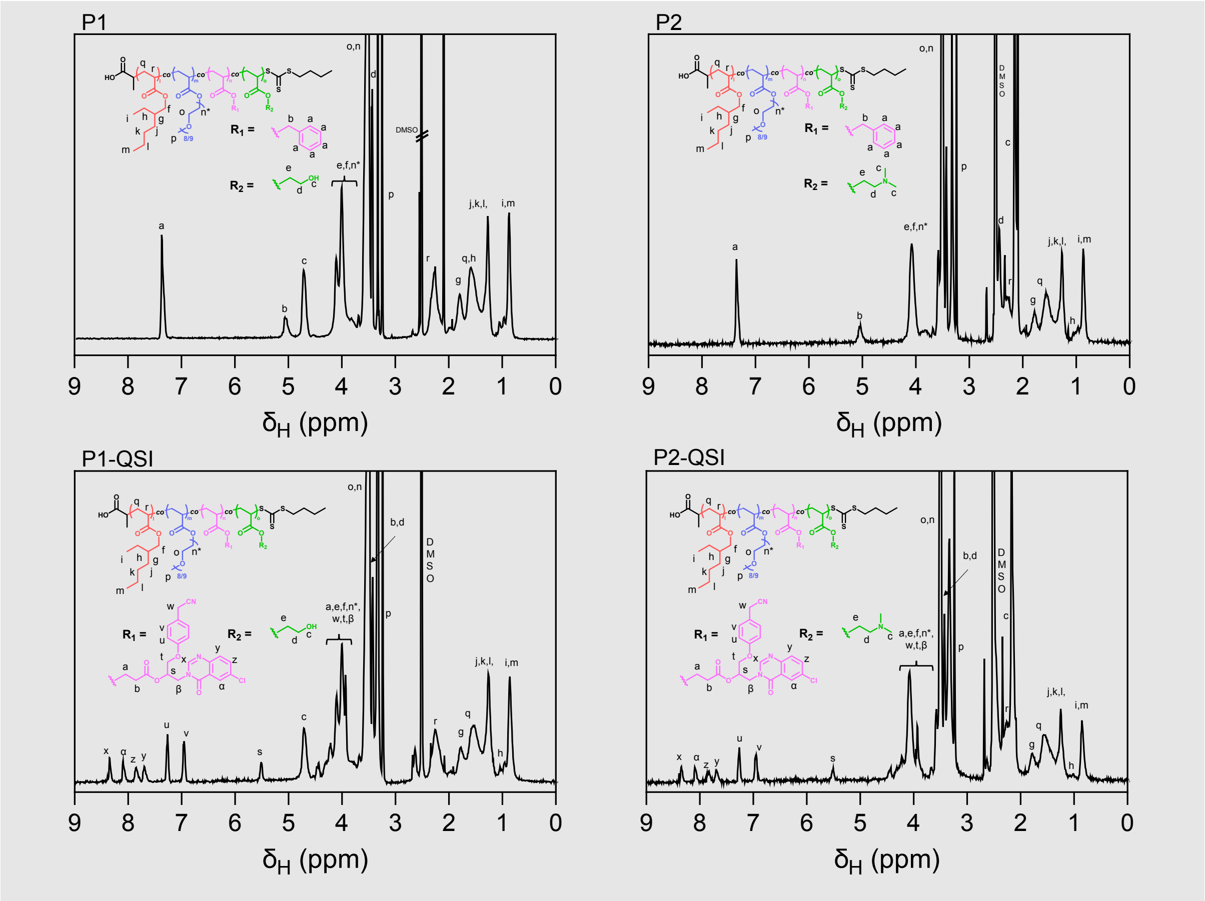

Figure S1.^{1}H NMR spectra of P1, P2, P1-QSI and P2-QSI after purification in DMSO-d_6.

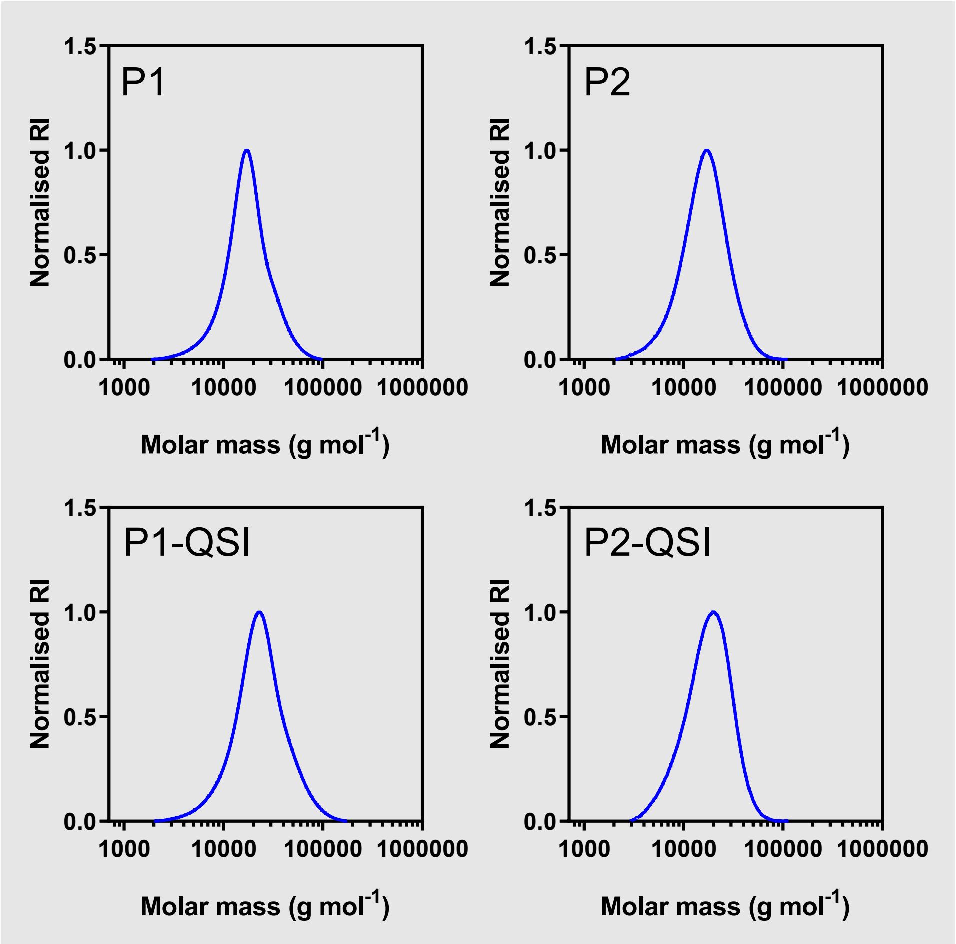

Figure S2. DMF-SEC chromatograms of P1, P2, P1-QSI and P2-QSI.

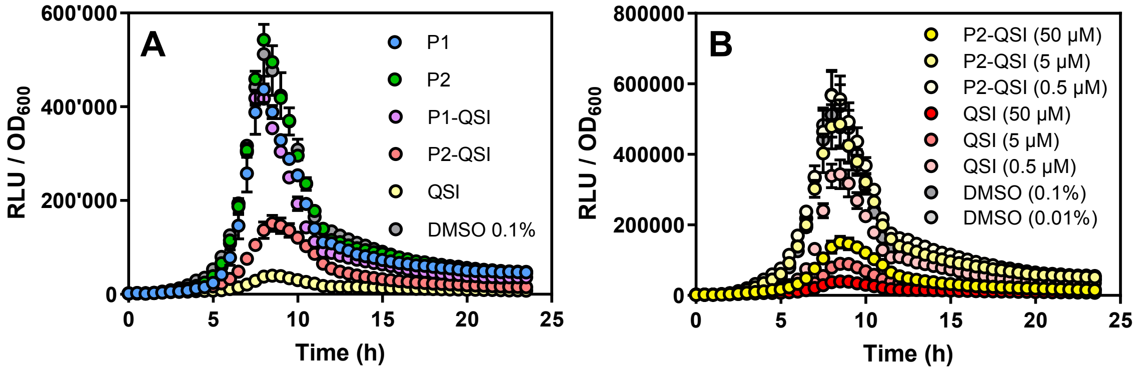

Figure S3. Effect of QSI and QSI polymers on a P_{pqsA}-*lux* transcriptional fusion in PAO1-L, which reports the PQS-dependent activation of the *pqs* operon mediated by PqsR. A) Comparison of interference with the PQS system in PAO1-L by polymers P1 and P2 at 50 µM and QSI at 10 µM. B) Inhibition of the *pqs* promoter (P_{pqsA}) activity in response to P2-QSI supplemented at 0.5 µM to 50 µM relative to QSI treatment. Values given are averages from three different cultures ± standard deviation and correspond to the relative light units normalised to culture density (RLU/OD_{600}) over time (24 h).

1. Gurnani, P.; Blakney, A. K.; Terracciano, R.; Petch, J. E.; Blok, A. J.; Bouton, C. R.; McKay, P. F.; Shattock, R. J.; Alexander, C., The <i>In Vitro</i>, <i>Ex Vivo</i>, and <i>In Vivo</i> Effect of Polymer Hydrophobicity on Charge-Reversible Vectors for Self-Amplifying RNA. *Biomacromolecules* **2020,** *21* (8), 3242-3253.
2. Soukarieh, F.; Liu, R.; Romero, M.; Roberston, S. N.; Richardson, W.; Lucanto, S.; Oton, E. V.; Qudus, N. R.; Mashabi, A.; Grossman, S.; Ali, S.; Sou, T.; Kukavica-Ibrulj, I.; Levesque, R. C.; Bergström, C. A. S.; Halliday, N.; Mistry, S. N.; Emsley, J.; Heeb, S.; Williams, P.; Cámara, M.; Stocks, M. J., Hit Identification of New Potent PqsR Antagonists as Inhibitors of Quorum Sensing in Planktonic and Biofilm Grown Pseudomonas aeruginosa. *Frontiers in Chemistry* **2020,** *8*.
3. Singh, N.; Romero, M.; Travanut, A.; Monteiro, P. F.; Jordana-Lluch, E.; Hardie, K. R.; Williams, P.; Alexander, M. R.; Alexander, C., Dual bioresponsive antibiotic and quorum sensing inhibitor combination nanoparticles for treatment of Pseudomonas aeruginosa biofilms in vitro and ex vivo. *Biomaterials Science* **2019,** *7* (10), 4099-4111.
